# Communication skills of medical students: Evaluation of a new communication curriculum at the University of Augsburg

**DOI:** 10.3205/zma001681

**Published:** 2024-06-17

**Authors:** Giulia Zerbini, Philipp Reicherts, Miriam Reicherts, Nina Roob, Pia Schneider, Andrea Dankert, Sophie-Kathrin Greiner, Martina Kadmon, Veronika Lechner, Marco Roos, Mareike Schimmel, Wolfgang Strube, Selin Temizel, Luise Uhrmacher, Miriam Kunz

**Affiliations:** 1 University of Augsburg, Faculty of Medicine, Department of Medical Psychology and Sociology, Institute of Theoretical Medicine, Augsburg, Germany; 2 University of Augsburg, Faculty of Medicine, Medical Education Sciences (DEMEDA), Augsburg, Germany; 3 University Hospital Augsburg, Psychooncology Service, CCCA, Augsburg, Germany; 4 University of Augsburg, Faculty of Medicine, Department of Psychiatry, Psychotherapy, and Psychosomatics, Augsburg, Germany; 5 University of Augsburg, Faculty of Medicine, Deanery, Augsburg, Germany; 6 University of Augsburg, Faculty of Medicine, Department of General Practice, Augsburg, Germany; 7 University of Augsburg, Faculty of Medicine, Department of Pediatrics and Adolescent Medicine, Augsburg, Germany; 8 University of Augsburg, Faculty of Medicine, Department of Hygiene and Environmental Medicine, Augsburg, Germany; 9 University of Augsburg, Faculty of Medicine, Department of Hematology and Oncology, Augsburg, Germany

**Keywords:** communication, empathy, education, communication skills, medical curriculum, simulated patients

## Abstract

**Objectives:**

Teaching communication skills plays a pivotal role in medical curricula. The aim of this article is to describe and evaluate a new communication curriculum developed at the Faculty of Medicine, University of Augsburg (KomCuA), which was conceptualized by an interdisciplinary team based on recommended quality standards (i.e., helical, integrated, longitudinal).

**Methods:**

A total of 150 medical students enrolled in the 1^st^, 3^rd^, and ≥5^th^ semester participated in the study. They completed an online survey (numerical rating scales and validated questionnaires) evaluating their current communication skills, how these developed across the curriculum in terms of quality and self-confidence, and how helpful they considered practicing in small group tutorials with simulated patients. The students’ attitudes towards communication and empathy in the context of medical care were additionally assessed. The students’ responses were compared across semesters using one-way univariate analysis of variance (ANOVA).

**Results:**

Overall, students reported improved communications skills due to attending the KomCuA and further considered practicing with simulated patients as being very helpful (large effect sizes). Compared to 1^st^ semester students, 3^rd^ and ≥5^th^ semester students reported better communication skills (medium to large effect sizes). Additionally, ≥5^th^ semester students showed stronger agreement towards the relevance of empathy in the context of medical care (medium effect size) compared to both 1^st^ and 3^rd^ semester students.

**Conclusion:**

The KomCuA has shown to be an effective communication curriculum to support medical students in the development of their communication skills and positive attitudes towards empathy. Additional studies assessing students’ communication skills and empathic attitudes longitudinally are warranted to confirm the present results and to gain further knowledge on how these essential skills and attitudes develop across medical curricula.

## Zusammenfassung

**Zielsetzungen:**

Die Vermittlung kommunikativer Kompetenzen spielt in der medizinischen Ausbildung eine zentrale Rolle. Ziel dieses Artikels ist es, ein neues Kommunikationscurriculum an der Medizinischen Fakultät der Universität Augsburg (KomCuA) zu beschreiben und zu evaluieren, welches von einem interdisziplinären Team basierend auf empfohlenen Qualitätsstandards (d.h. helikal, integriert, longitudinal) konzipiert wurde.

**Methoden:**

An der Online-Studie nahmen insgesamt 150 Medizinstudierende aus den Semestern 1., 3. und ≥5. teil. Mithilfe numerischer Bewertungsskalen und validierter Fragebögen wurden Aspekte wie Kommunikationskompetenz, Veränderungen in Qualität und Sicherheit der eigenen Kommunikation und Nutzen der Übungen mit Schauspielpatient*innen erfasst. Zusätzlich wurden die Einstellungen der Studierenden zu Kommunikation und Empathie im Kontext der medizinischen Versorgung bewertet. Die Antworten der Studierenden wurden mithilfe von Varianzanalysen (ANOVA) über die Semester hinweg verglichen.

**Ergebnisse:**

Insgesamt gaben die Studierenden an, dass sich ihre Kommunikationskompetenz durch die Teilnahme am KomCuA verbessert habe, und bewerteten das Üben mit Schauspielpatient*innen als sehr hilfreich (große Effektstärken). Im Vergleich zu den Studierenden des 1. Semesters berichteten die Studierenden des 3. und ≥5. Semesters über bessere Kommunikationskompetenzen (mittlere bis große Effektstärken). Die Relevanz von Empathie im Kontext der medizinischen Versorgung (mittlere Effektstärke) wurde von den Studierenden des ≥5. Semesters höher eingestuft als von Studierenden des 1. und 3 Semesters.

**Schlussfolgerung:**

Das KomCuA hat sich als wirksames Kommunikationscurriculum erwiesen, das sowohl die Entwicklung von Kommunikationskompetenzen bei Medizinstudierenden befördert als auch zu einer positiven Einstellung zur Rolle von Empathie beiträgt. Weitere Studien, in denen Kommunikationskompetenzen und empathischen Einstellungen der Studierenden im Längsschnitt untersucht werden, sind notwendig, um die vorliegenden Ergebnisse zu bestätigen und weitere Erkenntnisse darüber zu gewinnen, wie sich Kommunikation und Einstellungen im Laufe des Studiums entwickeln.

## 1. Introduction

### 1.1. Theoretical background

The importance of communication skills and an empathic attitude for the development of a trustful doctor-patient relationship have long been established, as well as their positive effects on the patients’ health and satisfaction with medical care [1], [2]. Given this importance, medical faculties have implemented teaching of communication as a fundamental skill in their curricula over the past years. Besides the obvious relevance of implementing communication trainings in medical curricula, it is important to evaluate their effectiveness. Previous studies mainly relied on surveys or exam results to assess the students’ attitudes towards the relevance of a good doctor-patient communication and to assess how communication skills improve [3], [4], [5]. Overall, teaching of communication skills has shown positive effects [6], [7], [8], [9], especially when being implemented in longitudinal curricula (vs. single courses), which allow students to repeatedly practice and refine their acquired communication skills [10], [11]. In general, students agree that communication skills are essential attributes of physicians, they report positive learning experiences, and they perform better during objective structured clinical examinations (OSCEs) of communication skills after having attended communication curricula [3], [4], [5], [12]. 

### 1.2. The new communication curriculum (KomCuA) at the Faculty of Medicine, University of Augsburg

**Figure 1:**
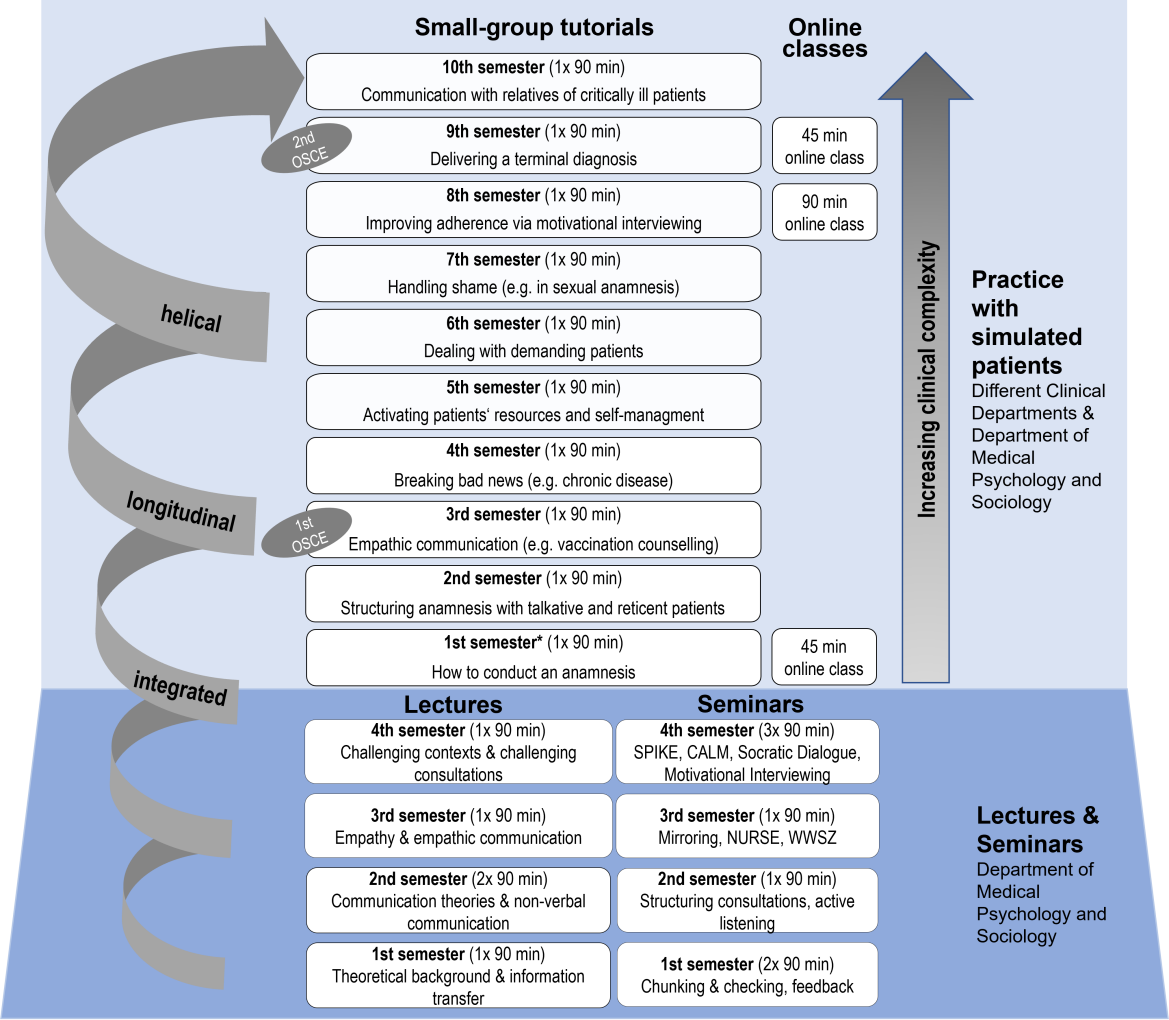
Overview of the communication curriculum of the Faculty of Medicine, University of Augsburg (KomCuA). Communication theories and techniques (5 lectures and 7 seminars for a total of 18 hours) are taught during the first 2 years (1^st^-4^th^ semester). Starting from the 2^nd^ semester, the students practice the acquired communication skills and techniques with SPs (9 small group tutorials complemented by 3 online classes for a total of 18 hours). The students’ communication skills are examined during two OSCEs (3^rd^ and 9^th^ semester). Note: *the 1^st^ semester students participate in a small group tutorial in which they practice history taking without SPs.

In October 2019, the first cohort of students started their medical training at the newly founded Faculty of Medicine, University of Augsburg (Germany). The goal and aspiration of this new medical degree program is to provide a professionally sound education, with particular emphasis on physician competencies and roles (including communication skills and empathic attitudes) [13]. Under the lead of the Department of Medical Psychology and Sociology, a new communication curriculum (Kommunikation Curriculum Augsburg; KomCuA) was designed and implemented in collaboration with the Departments of Psychiatry, Psychotherapy and Psychosomatics, Hematology and Oncology, General Practice, Pediatrics and Adolescent Medicine, Hygiene and Environmental Medicine, Gynecology, Urology, Otolaryngology, Head and Neck Surgery, Anesthesiology and Operative Intensive Care, Gastroenterology, Medical Education Sciences and the Psychooncology Service of the University Hospital Augsburg (see figure 1 (Fig. 1)). The fact that the Faculty of Medicine in Augsburg was newly founded offered the unique opportunity to design an interdisciplinary communication curriculum that was embedded and integrated within the overall medical curriculum from the start. Prior to developing the teaching contents of the curriculum, the interdisciplinary team met several times to set the goals and objectives of the KomCuA by defining the physician roles and communication competencies that are relevant across medical disciplines and that are also based on the National Competence-Based Catalogue of Learning Objectives for Undergraduate Medical Education (NKLM). Defining clear goals and objectives is an important step in the development of curricula as proposed by Thomas and colleagues (problem identification, targeted need assessment, goals and objectives, educational strategies, implementation, evaluation and feedback) [14]. The KomCuA follows the quality standards (i.e., helical, integrated, longitudinal curriculum) recommended by Silverman [15]. Communication skills are trained as a central rather than an add-on medical skill by integrating them with other clinical competencies and by allowing students to continuously develop, review and refine their skills by virtue of the longitudinal and helical structure of the curriculum. The KomCuA underwent intensive internal evaluations and revisions considering inputs from instructors, simulated patients (SPs) and students. It comprises a total of 36 hours of classes using various teaching formats (educational strategies): plenary lectures (all students), seminars (ca. 20 students), small group tutorials (10-12 students) and online classes (all students). The plenary lectures (5 in total) are grouped together at the beginning of the curriculum (first 4 semesters) to give the students a solid theoretical basis on the topics of physician-patient communication and relationship. The lectures are complemented by practical exercises on interviewing techniques, video analyses and discussions in seminar groups (7 seminars). The aim of the seminars is to give the students the possibility to deepen and reflect on the lectures’ contents and to also start practicing the learned communication techniques. The seminars have thus the important goal of bridging theory (lecture) and practical experience (small group tutorials with SPs). Already starting from the 2^nd^ semester, the students practice the acquired communication techniques with SPs in small groups, supervised by experienced instructors (9 tutorials). The small group format is embedded in the clinical longitudinal curriculum where central clinical practical skills are taught (e.g., physical examination, venipuncture). All tutorials with SPs are developed (including teaching material and scripts) in collaboration with the different clinical departments by a small interdisciplinary core team (psychologists and researchers, a psychotherapist, and a theater pedagogue; MK, GZ, PR, MR, NR). This ensures conceptual stability, with each session building upon previously taught concepts and skills and hereby reinforcing previous learning with increasing levels of difficulty and clinical complexity. Instructors, SPs and students are trained on how to provide a precise and constructive feedback to maximize the students’ learning experience [16]. Moreover, extensive trainings with the SPs and a joint rehearsal with SPs and instructors before each class (instructors playing the role of the doctor) ensures high quality and comparability across groups. Each tutorial starts with a brief repetition of the relevant theories and communication techniques (10 minutes), followed by practical exercises to “activate” the students (10 minutes). After that, at least two simulated consultations are carried out with two different SPs, to allow for a more diverse experience and to give plenty of opportunities (18 simulated consultations for each small group tutorial across the entire medical degree) to practice with the SPs. However, given that attendance is not mandatory for small group tutorials at the Medical Faculty in Augsburg, students might decide not to benefit from this training opportunity. After each consultation (7-10 minutes), detailed feedback is provided, starting with the self-evaluation of the student in the doctor role, followed by feedbacks from the SP, the peers and finally from the instructor (about 30 minutes). From the 5^th^ semester on, the simulated consultations take place in the skills lab of our medical faculty, offering a stronger resemblance to clinical settings and thus, a more ecological-valid context, as well as the possibility to observe the consultation from an enclosed room via a two-way mirror and audio transmission. Up to semester 4, the tutorials take place in regular seminar rooms.

In summary, the main features of the KomCuA are the following: the early incorporation of the communication curriculum in the development of the entire medical curriculum, the conceptualization and implementation of the curriculum under the lead of a core team, the interdisciplinary collaboration with several clinics, the intensive training and preparation dedicated to the tutorials with the SPs, the integrated, helical, and longitudinal structure of the curriculum as well as its scientifical evaluation. Since October 2019 the curriculum is in the implementation and evaluation/feedback phase.

### 1.3. Objectives of the study 

The aim of this study was to evaluate the KomCuA by assessing whether the communication skills and the students’ attitudes towards communication and empathy differ across semesters (1^st^, 3^rd^, ≥5^th^) and by asking the students to evaluate whether SPs are helpful to learn communication skills and techniques.

We hypothesized that ≥5^th^ semester students would report better communication skills compared to 3^rd^ semester students and that 3^rd^ semester students would report better skills compared to 1^st^ semester students. Similarly, we expected the attitudes towards communication and empathy to be more positive with increasing semester. Based on previous studies, we also expected the students (independent of semester) to report overall positive experiences with the SPs. 

## 2. Methods

Data were collected with an online survey at the beginning of the winter semester 2022/2023 (cross-sectional design). The study was approved by the responsible ethics committee of the University of Munich, Germany (project number 22-0659) and was conducted in accordance with the Declaration of Helsinki.

### 2.1. Participants 

Medical students at the University of Augsburg enrolled in the 1^st^, 3^rd^, 5^th^, or 7^th^ semester were informed and recruited to participate in the study via internal emails. All participants gave their written informed consent and received course credit (0.5 “Versuchspersonenstunde”) or monetary compensation (5 Euros) for their participation. At the time of the survey, 1^st^ semester students had not yet attended any of the communication curriculum classes, 3^rd^ semester students had attended 3 lectures, 3 seminars, 1 online class, and 2 small-group tutorials, and 5^th^/7^th^ semester students had attended 5 lectures, 7 seminars, 1 online class and 4/6 small group tutorials.

### 2.2. Online survey 

The online survey included single items (adapted from Zimmermann et al. [5]) and validated questionnaires with good psychometric properties to assess the students’ self-reported communication skills, to evaluate the use of SPs, as well as to assess the students’ attitudes towards communication and empathy in the context of medical care. The survey was conducted using the software SoSci Survey ([soscisurvey.de]; SoSci Survey GmbH, Munich, Germany).

#### 2.2.1. Communication skills and curriculum evaluation

**Figure 2:**
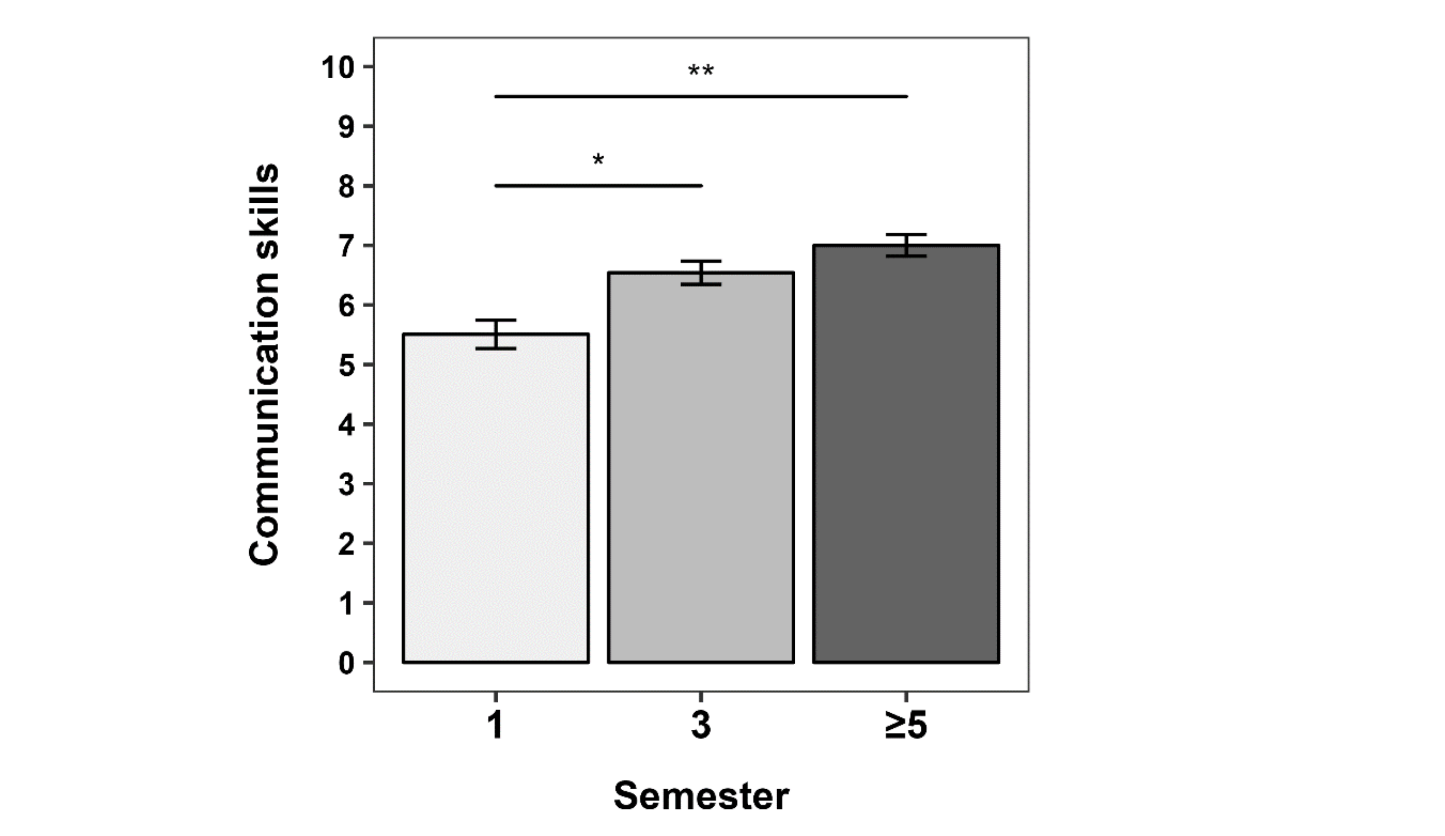
Communication skills The mean self-reported communication skill scores of the students from semesters 1, 3 and ≥5 are plotted. The following item was used: “How would you rate your communication skills in the context of doctor-patient communication?” with response options on a 11-point numerical rating scale from 0 (very bad) to 10 (very good). Error bars indicate the standard error of the mean (SEM). *p<.016, **p<.001 (α level adjusted at 0.016 for multiple testing).

**Figure 3:**
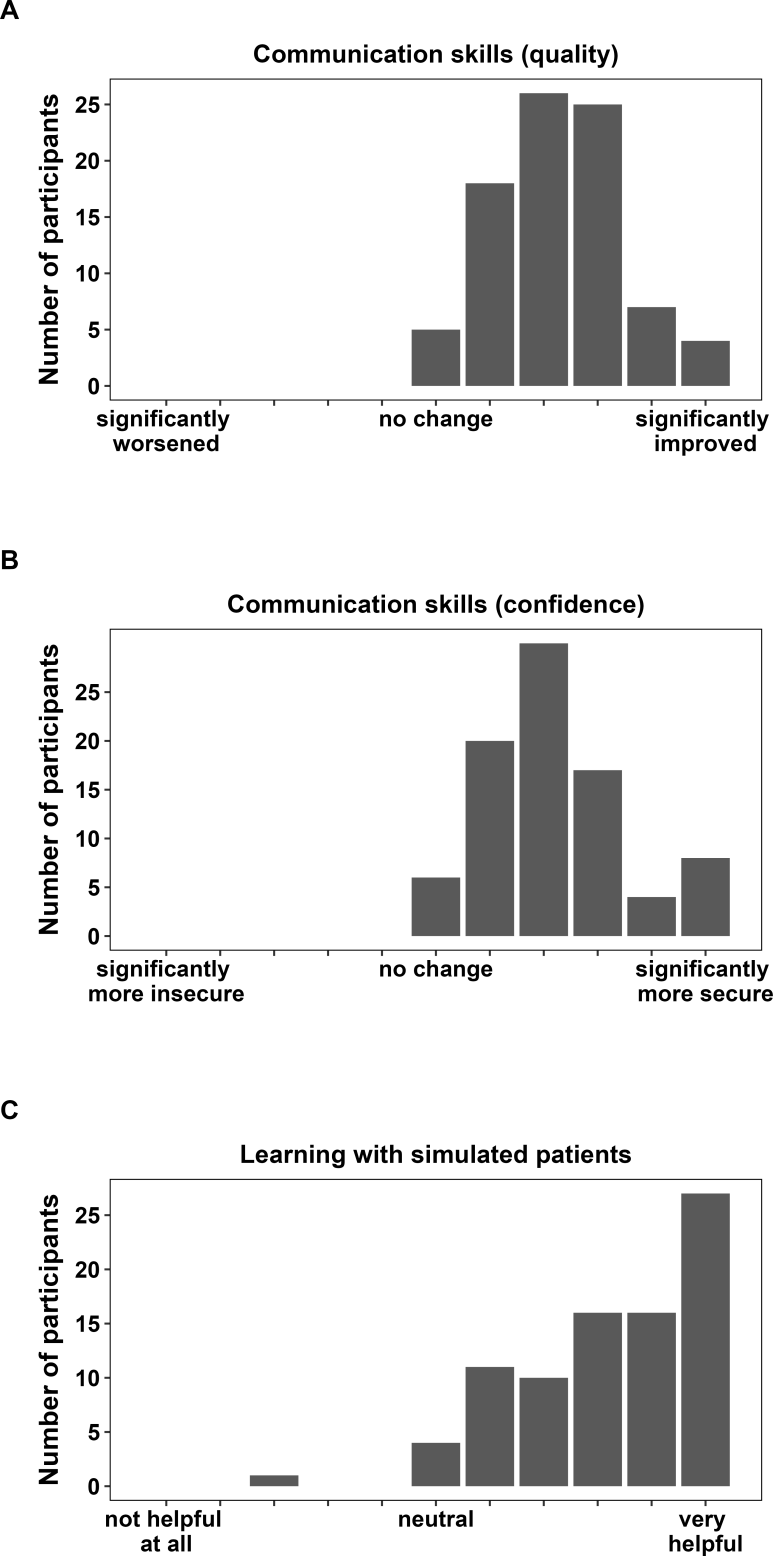
Communication skills (change in quality and confidence) and learning with SPs A) Distribution of the students’ responses regarding a change of their communication skills (quality) due to attending the communication curriculum. The following item was used: “How would you rate the change in your communication skills as a result of attending the communication curriculum?”, with response options on a 11-point numerical rating scale from 0 (significantly worsened), 5 (no change) to 10 (significantly improved). B) Distribution of the students’ responses regarding a change of their communication skills (confidence) due to attending the communication curriculum. The following item was used: “How would you rate the change in your communication skills as a result of attending the communication curriculum?”, with response options on a 11-point numerical rating scale from 0 (significantly more insecure), 5 (no change) to 10 (significantly more secure). C) Distribution of the students’ responses regarding the usefulness of practicing with SPs. The following item was used: “Learning communication skills with simulated patients is…” with response options on a 11-point numerical rating scale from 0 (not helpful at all), 5 (neutral) to 10 (very helpful). Notes: Data derive only from 3^rd^ and ≥5^th^ semester students because 1^st^ semester students had not yet attended any communication teaching at the time of the survey.

We used 11-point numerical rating scales to quantify the students’ self-perception of their own communication skills (all semesters), the perceived change of their own communication skills (quality and self-confidence) due to attending the communication curriculum (all semesters except for the 1^st^), and the perceived usefulness of practicing with SPs (all semesters except for the 1^st^). These items were adapted from Zimmermann et al. [5] (for more details on the items used here see the captions of figure 2 (Fig. 2) and figure 3 (Fig. 3)).

#### 2.2.2. Attitudes towards communication skills (CSAS-G) and empathy (JSPE-S)

We used the German version of the Communication Skills Attitude Scale (CSAS-G; [17]) to assess the students’ attitudes towards the importance of learning and implementing communication skills in a doctor-patient setting. The scale has a total of 26 items with response options ranging from 1 (strongly disagree) to 5 (strongly agree), which load on two factors (positive (CSAS-PAS) and negative (CSAS-NAS) communication attitudes).

We used the German student version of the Jefferson Scale of Physician Empathy (JSPE-S; [18], [19]) to assess students’ perceived relevance of empathy in the context of specific patient care situations. The JSPE-S has 20 items with response options ranging from 1 (strongly disagree) to 7 (strongly agree). The total score was calculated by summing all items.

### 2.3. Statistical analyses

Statistical analyses were performed using R software (version 4.0.5 [20]). The dependent variables (communication and empathy attitudes/skills) were compared with one-way univariate analysis of variance (ANOVA) across the different semesters (3 factor levels). Sample sizes for each semester varied, especially sample sizes in semesters 5 and 7 were smaller compared to those in semesters 1 and 3. Given that 5^th^ and 7^th^ semester students were similar regarding their training status (number of lectures and seminars is the same, only number of small group tutorials differs: 4 vs. 6) and because they did not significantly differ in terms of self-reported communication skills and attitudes towards empathy and communication (all p-values >.05), these students were merged into one group (≥5^th^ semesters). In case of a significant main effect, post hoc tests were run to identify which semesters significantly differed with respect to the dependent variables of interest (Bonferroni correction with the α level adjusted at 0.016 was applied for multiple testing). One-sample t-tests (reference value=5, corresponding to “no change” on a scale 0-10) were run to determine whether the students (3^rd^ and ≥5^th^ semesters) reported an improvement in their communication skills and whether they considered practicing with SPs as being helpful. Given that gender was not significantly associated with any dependent variable of interest, it was not added to any of the models as covariate. 

## 3. Results

### 3.1. Demographics

A total of 352 students were enrolled at the Faculty of Medicine when the study took place, and 187 students took part in the study. 37 of them (20%) did not fill in all the questionnaires resulting in a sample of 150 students with a complete dataset (95 females, 54 males, 1 non-binary, mean age: 21.8±SD 3.2 years). 65 students were enrolled in the 1^st^ semester, 48 students in the 3^rd^ semester and 37 students in the ≥5^th^ semesters (26 in the 5^th^ and 11 in the 7^th^ semester). The students reported to have attended on average 85% of the classes (course attendance was not mandatory). 

### 3.2. Online survey

Average scores (±SD) of all questionnaires across semesters are reported in table 1 (Tab. 1). 

#### 3.2.1. Communication skills

The students’ self-perception of their own communication skills differed significantly between semesters (one-way ANOVA; F_2,147_=12.130, p<.001; see figure 2 (Fig. 2)). Post-hoc comparisons revealed that communication skills were rated significantly higher (6.5 vs. 5.5 on a scale 0-10) by 3^rd^ compared to 1^st^ semester students (p=0.001; Cohen’s d=0.6). The self-reported communication skills of students from the ≥5^th^ semesters were descriptively higher compared to both 1^st^ and 3^rd^ semesters, but only the comparison with the 1^st^ semester students was significant (7.0 vs. 5.5 points; p<.001; Cohen’s d=0.9). 

Students in the 3^rd^ and ≥5^th^ semesters were additionally asked to evaluate whether the curriculum had an influence on their communication skills and whether the practice with SPs was helpful (see figure 3 (Fig. 3)). One-sample t-tests showed that the students reported a significant improvement in their communication skills, both in terms of quality (on average 7.2 on a 11-point scale with 5=no change; t(84)=17.458, p<.001; Cohen’s d=1.89) and confidence (on average 7.2 on a 11-point scale with 5=no change; t(84)=15.406, p<.001; Cohen’s d=1.67). Moreover, the tutorials with SPs were rated as being very helpful (on average 8.2 on a 11-point scale with 5=neutral; t(84)=17.455, p<.001; Cohen’s d=1.89). 

#### 3.2.2. Attitudes towards communication skills and empathy

One-way ANOVAs were performed to investigate whether students’ attitudes towards communication skills in a medical setting (CSAS positive and negative subscales) changed across semesters. There was a statistically significant main effect of semester only for the positive subscale (F_2,147_=4.654, p=0.011). Post-hoc comparisons between semesters showed that 1^st^ semester students reported more positive attitudes compared to 3^rd^ semester students (p=0.003; Cohen’s d=0.6). The attitudes of ≥5^th^ semester students were similar to those of 1^st^ semester students and descriptively (but not significantly) higher compared to 3^rd^ semester students (p=0.053; Cohen’s d=0.4). No further post-hoc comparisons were significant.

**Figure 4:**
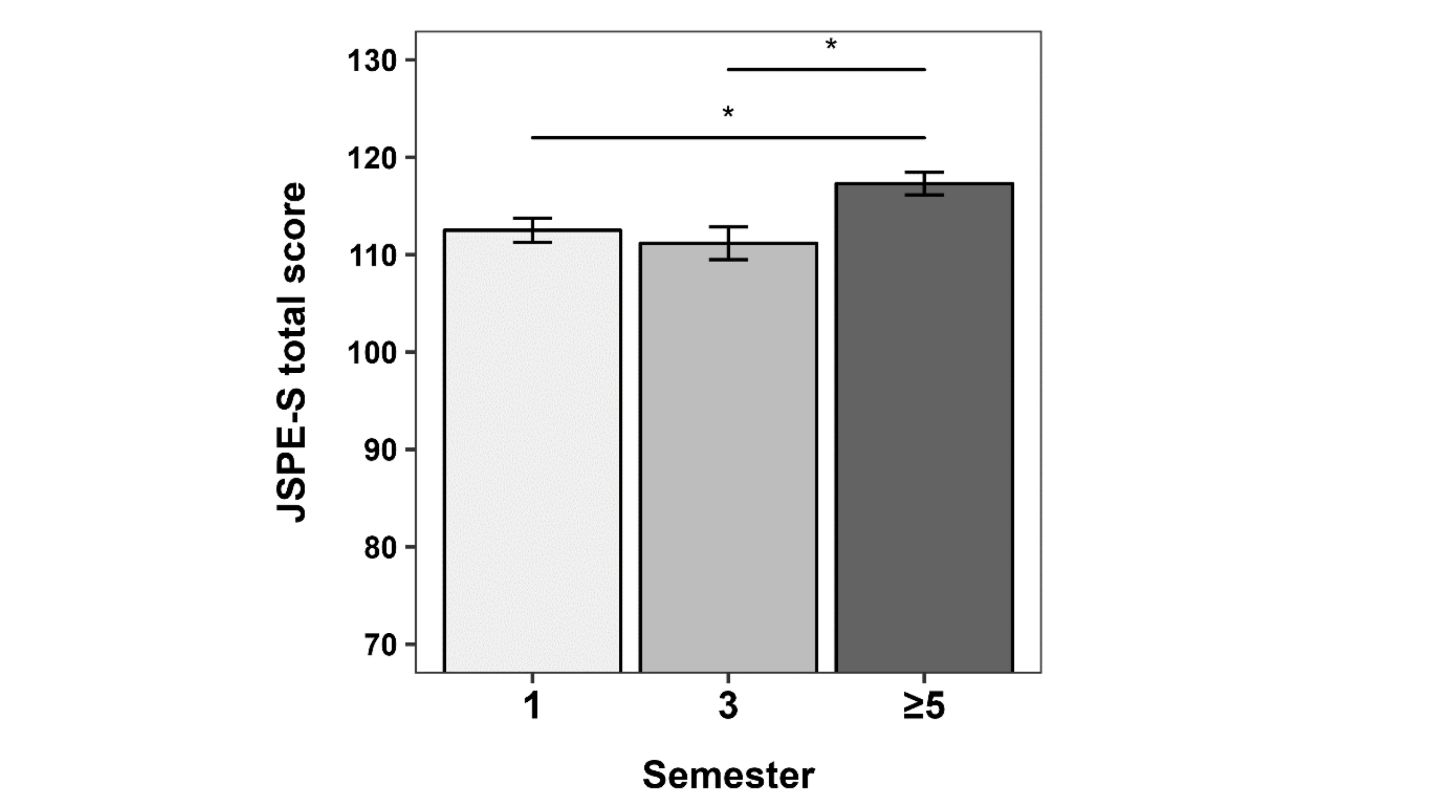
Attitudes towards empathy in the context of medical care We used the German student version of the Jefferson Scale of Physician Empathy (JSPE-S) to assess students’ perceived relevance of empathy in the context of specific patient care situations. The mean total JSPS-S scores of the students from semesters 1, 3 and ≥5 are plotted (possible range score is 20-140 with higher scores indicating more positive attitudes towards empathy). Error bars indicate the standard error of the mean (SEM). *p<.016 (α level adjusted at 0.016 for multiple testing).

Regarding the students’ attitudes towards empathy in the medical care context, we found a statistically significant effect of semester (F_2,147_=4.327, p=0.015; see figure 4 (Fig. 4)). ≥5^th^ semester students scored significantly higher on the JSPE-S compared to both 1^st^ semester (p=0.006; Cohen’s d=0.5) and 3^rd^ semester students (p=0.004; Cohen’s d=0.6). No further post-hoc comparisons were significant. 

## 4. Discussion

The main aim of this article was to describe and evaluate the KomCuA, a newly developed communication curriculum at the Faculty of Medicine, University of Augsburg. To this end, the communication skills and the attitudes towards communication and empathy of students enrolled in the 1^st^ semester, who had not yet started the communication curriculum, were compared to students enrolled in the 3^rd^ and in the ≥5^th^ semesters. Students were additionally asked to evaluate how their communication skills changed due to attending the communication curriculum and how helpful they perceived practicing with SPs.

### 4.1. KomCuA evaluation

Our results show that the KomCuA has a positive impact on students’ reported communication skills. 3^rd^ and ≥5^th^ semester students reported better communication skills compared to 1^st^ semester students (on average 1.0 and 1.5 points higher, respectively, on a scale ranging from 0-10; medium to large effect sizes). Differences in communication skills between 1^st^ and 3^rd^ semester students were more pronounced compared to 3^rd^ and ≥5^th^ semester students, which could be due to more room for improvement at the beginning of the curriculum. Overall, students also reported that their communication skills improved in terms of both quality and confidence because of attending the communication curriculum, and they similarly evaluated the possibility of practicing with SPs as (very) helpful (large effect sizes). Our findings are in line with previous studies that evaluated communication curricula at other medical faculties and that reported positive evaluations by students [3], [4], [5]. It is important to note that the KomCuA has no mandatory attendance; nevertheless, students attended on average 85% of the curriculum classes, which per se indicates a fundamental interest in learning and practicing communication skills as well as a perceived benefit of the training. We could not yet evaluate the entire KomCuA because our oldest cohort of students was enrolled in the 7^th^ semester at the time of the survey. However, given the positive effects observed here and the helical, integrated, longitudinal nature of our curriculum, we expect even stronger effects when students proceed in their studies.

#### 4.1.1. Attitudes towards communication skills and empathy

Besides asking students to rate their own communication skills and whether these changed due to our communication curriculum, we also assessed students’ attitudes towards the importance of communication skills in a doctor-patient setting. Here, however, we did not find clear differences across semesters. The positive attitudes towards the importance of communication skills were significantly lower among 3^rd^ compared to 1^st^ semester students; however, they increased again in the higher semesters (not significantly different from semester 1). Previous studies using the same questionnaire (CSAS) have also found inconsistent results, with the students’ attitudes towards communication skills changing in both directions (increasing and decreasing) after attending a communication training or curriculum [17], [21], [22], [23]. Considering that the positive attitude scores were already high among 1^st^ semester students (on average 51.3 with a score range of 13-65) there was only a small margin of improvement along with the training, which could explain why the attitudes of older students were not significantly more positive compared to the younger ones. Similarly, the negative attitude scores were low for all students (on average 24.5 with a score range of 13-65), indicating again a limited possibility of change by semester. 

Regarding the attitudes towards the relevance of empathy in the context of medical care, we found a positive impact of our curriculum, with the ≥5^th^ semester students rating the relevance of empathy significantly higher compared to 1^st^ and 3^rd^ semester students. The absence of a significant difference between 1^st^ and 3^rd^ semester students might be explained with the organization of the teaching contents of our curriculum. The relevance of empathy is discussed in detail in the 3^rd^ semester and deepened in the 4^th^ semester, meaning that the 3rd semester students just had a first introduction into this topic at the time of the survey. Our results are in contrast with previous findings describing a decline of medical students’ empathy across the medical degree [24], [25], [26]. A recent review, however, concluded that findings are often inconsistent, with studies reporting lower, mixed, higher or unchanged levels of empathy across the medical degree [27]. A potential reason for the mixed findings might be that students, especially during the first years of studies, have usually limited contact with real patients, likely making the evaluation of the role of empathy somewhat hypothetical and thus imprecise. At our faculty, on the contrary, the first contact with real patients is very early on (already in the 1^st^ semester, but not necessarily with a main focus on communication skills or empathy).

### 4.2. Limitations

The cross-sectional design of our study limits the interpretation of the results in terms of causality. However, our findings across different variables consistently indicating better communication skills and empathic attitudes among the students in higher semesters point in the direction of a very likely positive influence of our curriculum. Another problem of cross-sectional designs are possible cohort effects. However, we assume that such effects are unlikely in our sample, given that the participants’ cultural and generational characteristics are very similar. Still, we did not collect detailed socio-demographic data (in addition to sex and age), which could have been helpful in substantiating this assumption. Finally, it is important to note that the reported improvement in communication skills is prone to recall biases, since we asked the students to retrospectively compare their communication skills at the beginning of their medical degree vs. at the time of data collection and we did not measure the students’ communication skills longitudinally.

## 5. Conclusions and outlook

Our findings show that the KomCuA is an effective communication curriculum that supports medical students in developing and improving their communication skills and empathic attitudes. As previously reported [28], the possibility of learning with SPs is considered by students to be a very positive and essential tool to practice communication techniques and to improve communication skills and thus, might be implemented in medical curricula right from the beginning. Attitudes towards the relevance of good communication and empathy in the medical care context were already positive among 1st semester students, leaving small room for improvement. Still, empathy was evaluated as more important by the ≥5^th^ semester students. Future studies should use longitudinal designs to further investigate how communication skills and empathic attitudes change and develop over time and especially across medical communication curricula.

## Data

Data for this article are available from Dryad Repository: [https://doi.org/10.5061/dryad.k6djh9wcj] [29]

## Authors

### First authorship

The authors Giulia Zerbini and Philipp Reicherts share the first authorship.

### Authors’ ORCIDs

- Giulia Zerbini: [0000-0002-5348-9212]
- Philipp Reicherts: [0000-0002-7031-0261]
- Pia Schneider: [0000-0002-8189-8961]
- Martina Kadmon: [0009-0007-0103-3567]
- Marco Roos: [0000-0003-1596-5908]
- Mareike Schimmel: [0000-0003-0611-3922]
- Wolfgang Strube: [0000-0003-2380-7651]
- Miriam Kunz: [0000-0002-0740-6738]

## Acknowledgements

We thank all the instructors and simulated patients involved in the KomCuA.

## Competing interests

Sophie-Kathrin Greiner is a member of the advisory board of the GOLDKIND Foundation. Wolfgang Strube has received a paid speakership from Mag & More (neurocare) and Recordati. He was member of an advisory board of Recordati.

The others authors declare that they have no competing interests.

## Figures and Tables

**Table 1:**
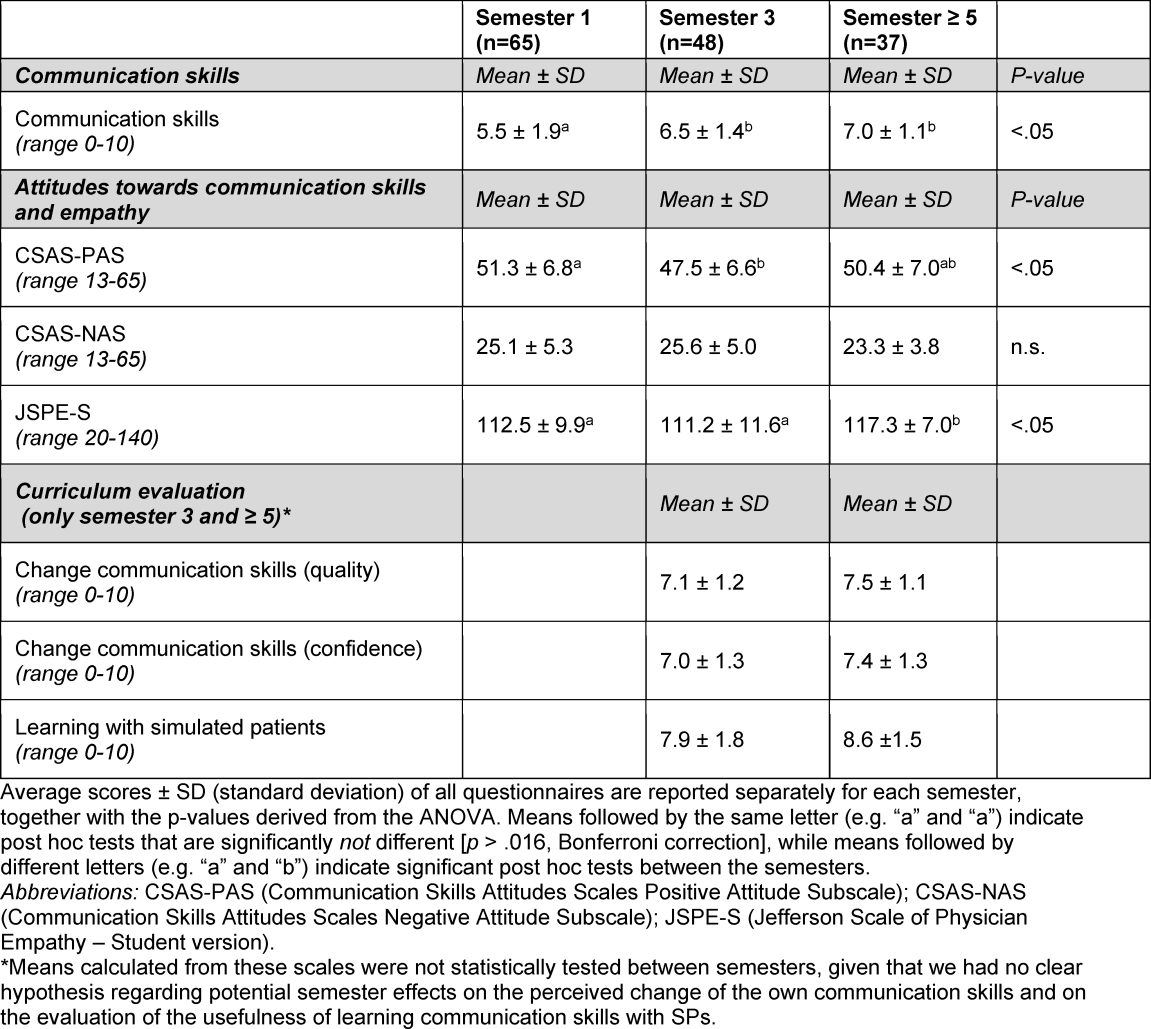
Overview of online survey questionnaires
